# Influence of Yield Stress and Material Area Ratio on Bondability and Formability in Drawing Processes of Bimetallic Rods

**DOI:** 10.3390/ma18071441

**Published:** 2025-03-25

**Authors:** Yeong-Maw Hwang, Hiu Shan Rachel Tsui

**Affiliations:** Department of Mechanical and Electro-Mechanical Engineering, National Sun Yat-sen University, Kaohsiung 804, Taiwan; ymhwang@mail.nsysu.edu.tw

**Keywords:** bimetallic rods, yield stress, core ratio, formability, drawing limit, bondability

## Abstract

Finite element simulations were conducted to investigate the drawing process of bimetallic rods, a key manufacturing technique used in aerospace, automotive, and advanced engineering applications. The study focused on how independent variations in the yield stress of the core and sleeve (150, 200, 250, 300 and 350 MPa) and differences in the initial core ratio (10%, 30%, 50%, 70% and 90%) affect bondability, formability, and fracture behavior. Simulations showed that the maximum achievable reduction ratio varied from approximately 50% to 55%, and hence, we focused on this range. By analyzing the maximum achievable reduction ratio and the distribution of effective strain, the simulations provided insights into the deformation mechanisms and failure modes of these composite structures. The results reveal that increasing the yield stress in either the core or the sleeve reduces the drawing limit by promoting stress concentrations at the interface, leading to premature failure and weakened bondability. Moreover, the core ratio critically influences performance: high core ratios result in thin, vulnerable sleeves prone to early fracture, while low core ratios produce thin cores that fail under high deformation loads. Strain analysis indicated that higher core yield stress increased interfacial shear stress, leading to localized failure, while a lower core yield stress resulted in more uniform material flow. A balanced core ratio (approximately 50%) yields a more uniform strain distribution, though it requires robust interfacial bonding to prevent delamination. These findings underscore the importance of optimizing both material properties and geometric configurations to enhance bondability, formability, and structural integrity during the drawing process of bimetallic rods.

## 1. Introduction

Bimetallic materials have attracted substantial attention due to their enhanced mechanical properties, cost-effectiveness, and multifunctionality in various engineering applications. Among these, bimetallic rods are extensively used in the aerospace, automotive, and manufacturing industries, where the combination of distinct materials enables tailored performance [1,2,3,4,5,6,7]. A key manufacturing process for such rods is drawing, in which the rod’s cross-sectional area is reduced via a sequence of dies. This process significantly influences formability, bondability, interfacial behavior, and final mechanical properties, thereby necessitating the optimization of material configurations and processing parameters for defect-free production and enhanced performance.

Despite these valuable insights into bimetallic rod deformation, few studies have specifically addressed bondability and forming limits during the drawing process. The interaction between core and sleeve materials—especially when there is a mismatch in mechanical properties—can lead to strain concentration and premature failure via mechanisms such as necking, delamination, and fracture. Therefore, a systematic investigation into these parameters is essential to improve the predictability of bimetallic rod manufacturing.

This study aims to extend the understanding of bimetallic rod drawing by examining the effects of yield stress variations in both the core and sleeve materials, as well as the initial thickness ratios, on formability and fracture behavior. Specifically, we investigate how yield stress influences the maximum achievable reduction ratio before failure (i.e., the drawing limit). In our simulations, both the outer and inner layers are modeled using yield stress values based on AISI-1006 low-carbon steel (the standard value is 250 MPa and the simulation values ranging from 150 to 350 MPa). Additionally, the effect of the core-to-sleeve thickness ratio (hereafter referred to as the “core ratio”), varied from 10% to 90%, is examined with respect to strain distribution and interfacial behavior. Finite element simulations are performed using DEFORM software (v11.0.2) under cold drawing conditions (drawing speed of 10 mm/s and a friction coefficient of 0.1, maintained via effective lubrication), thereby enabling a systematic evaluation of the parameters influencing strain distribution, failure modes, and maximum reduction ratio.

The outcomes of this research are expected to enhance our understanding of the mechanical behavior of bimetallic rods during drawing. By systematically investigating the influence of yield stress and thickness ratios on forming limits, this work aims to provide valuable guidelines for selecting optimal material combinations and process parameters. Moreover, the insights gained may facilitate further studies on advanced composite materials and multilayered metal structures, broadening their applications in emerging fields such as flexible electronics and biomedical devices.

## 2. Literature Review

Several studies have investigated different aspects of bimetallic wire drawing and deformation behavior. Szulc et al. [8] analyzed Cu–Steel bimetallic rods and found that the copper sleeve achieves a substantially higher reduction ratio compared to conventional steel drawing. Ko et al. [9] examined the effects of the initial outer layer proportion, die semi-angle, and reduction ratio on the final sleeve thickness, establishing that the initial outer layer proportion is the dominant factor influencing thickness distribution. Sasaki et al. [10] analyzed the microstructural evolution of Cu–Steel bimetallic wires, showing that core material differences significantly affect grain structure and hardness after drawing. Their FEM simulations revealed stress concentration at the interface, influencing mechanical response and failure behavior. Danenko et al. [11] explored the influence of drawing on the properties of bimetallic wire composed of carbon steel and 12X18H10T stainless steel. The results demonstrated that the deformation mechanism and interfacial stress significantly affect the final mechanical properties of the bimetallic wire. Chen et al. [12] examined the first-step drawing process of platinum-clad nickel bars, focusing on the effects of die semi-angle and cladding thickness on drawing force, equivalent stress, and interface behavior. The study validated FEM simulations with experimental results, reinforcing the applicability of numerical modeling in predicting deformation behavior.

The integrity of interfacial bonding plays a crucial role in determining the mechanical performance of bimetallic wires. Chen et al. [13] employed finite element analysis (FEA) to explore the deformation behavior of Al–Cu composite wires, revealing that fractures typically initiate at the interface where strain accumulation is maximal. These simulation findings, corroborated by experimental observations, underscore the role of interfacial strain localization in failure mechanisms. Malaki et al. [14] highlighted the crucial role of interfacial bonding in preventing delamination, emphasizing the need for proper surface treatments prior to processing. Their study showed that weak bonding between layers can lead to premature failure, particularly in applications requiring high mechanical reliability. Raghebi et al. [15] investigated the effect of interlayer pressure on Cu–Al bimetallic wire drawing, analyzing how die semi-angle and area reduction impact interface bonding quality. Their FEM simulations demonstrated that interlayer pressure varies significantly with processing conditions, affecting the final mechanical performance. Dai et al. [16] provided a theoretical analysis of the bimetallic bonding mechanism in Cu–Al clad wire drawing at room temperature. Their findings suggest that atomic interactions at the interface play a crucial role in bond strength, even in the absence of significant diffusion. Księżarek et al. [17] presented a study on the mechanical cold cladding technology for Fe/Cu and FeNi42/Cu bimetallic wires, focusing on the metallurgical bonding mechanism, microstructural evolution, and mechanical testing methods. The study highlighted the role of atomic diffusion and cold drawing in improving bond strength.

The microstructural evolution of bimetallic wires significantly influences their mechanical properties, especially after severe plastic deformation and heat treatment. Sasaki et al. [18] investigated the formation of ultra-fine copper grains (~200 nm) near the Cu/Al interface in drawn wires after heat treatment. These ultra-fine grains resulted from low-temperature recrystallization and enhanced interfacial strength. Keller et al. [19] examined the microstructure and mechanical properties of architectured Cu-Al composite wires manufactured through cold drawing. The study analyzed the effects of severe plastic deformation and heat treatment on grain refinement, strain hardening, and intermetallic compound formation, showing that heat-treated samples exhibited improved ductility due to recrystallization. Song et al. [20] evaluated the mechanical and electrical properties of a copper-clad aluminum (CCA) wire after a multistage cold drawing process followed by annealing. The study found that intermetallic compounds (Al_2_Cu, AlCu, and Al_4_Cu_9_) formed at the Cu/Al interface, influencing both mechanical integrity and electrical conductivity.

Beyond deformation studies, forming limit diagrams (FLDs) have been widely used to predict failure mechanisms in metal forming processes. Alberti et al. [21] utilized FEA to predict cracking conditions in UNI-3571 aluminum during drawing and validated their results experimentally, presenting the data in an FLD where fractured and non-fractured regions are delineated by an L-shaped identification curve. Similarly, McAllen and Phelan [22] evaluated the formability of aluminum Al–2011 through FEA and mapped their results onto an FLD. Haddi et al. [23] published the FLD for ETP 99% copper wire by simulating 16 combinations of half-mode angles and reduction rates—selecting two cases for experimental validation—which also resulted in an L-shaped curve. Roh et al. [24] investigated high-carbon chromium bearing steel (SUJ2Z) to determine the critical damage values under various half-mode angle and reduction rate combinations, subsequently plotting the corresponding FLD.

## 3. Materials and Methods

**Figure 1:**
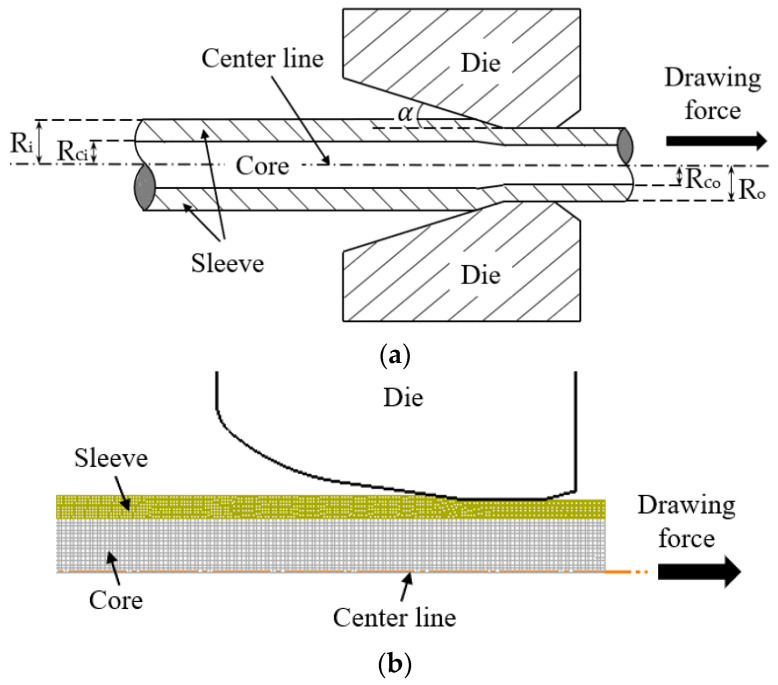
(**a**) Schematic diagram of bimetallic rod drawing; (**b**) simulation model of bimetallic rod drawing.

DEFORM (v11.0.2) is a finite element analysis software widely used for simulating metal forming processes. It predicts material behavior under various loading conditions by accounting for factors such as plasticity, strain rate, contact conditions, and friction. The software provides detailed information on stress, strain, and defect formation, thereby enabling an accurate analysis of material flow and tool wear. In this study, DEFORM was used to simulate and analyze the drawing process of bimetallic rods. The drawing process was examined through finite element simulations. Bimetallic rods—comprising a core and a sleeve—were modeled using an axisymmetric approach in DEFORM. Figure 1 presents a schematic diagram of the simulation model.

A rigid round die was modeled with an exit diameter of 10 mm and a semi-die angle of 8°. The semi-die angle of 8° was selected based on standard cold-drawing practices for steel-based bimetallic rods, particularly AISI-1006 with a yield stress of 250 MPa. While larger die angles are often recommended for softer materials, our study also considers higher yield stress conditions, where excessive die angles could increase interfacial shear stress, worsen deformation uniformity, and raise the risk of delamination. To accommodate this broader range of material strengths, we selected 8° as a balanced compromise, aligning with industrial practices and the median yield stress (250 MPa) among our tested conditions, ensuring stability and relevance across different material combinations.

The forming parameters used in the simulations are summarized in Table 1. The material properties, listed in Table 2, were selected to investigate the individual effects of the core’s and the sleeve’s yield stress on the formability of bimetallic rods. In this study, the yield stress of the core and sleeve were varied independently over the range of 150 to 350 MPa. To isolate the impact of yield stress variations, the Young’s modulus was maintained at 207 GPa for both layers. Additionally, a variety of core-to-sleeve thickness ratios were considered to assess their influence on strain distribution and interfacial behavior.

The reduction ratio was gradually increased until failure, with the forming limit defined as the maximum achievable reduction ratio prior to failure. This systematic investigation aims to provide insights into how the individual yield stresses of the core and sleeve affect the formability characteristics of bimetallic rods under various material and processing conditions, thereby guiding the optimization of manufacturing parameters.

## 4. Results

### 4.1. Influence of Core and Sleeve Yield Stress on Drawing Limit at a Fixed 70% Core Ratio

This section examines how variations in the yield stress of the core (σ_core_) and the sleeve (σ_sleeve_) affect the formability and bondability of bimetallic rods when the initial core ratio is fixed at 70%. In these simulations, both σ_core_ and σ_sleeve_ are independently varied over the range of 150 to 350 MPa.

**Figure 2:**
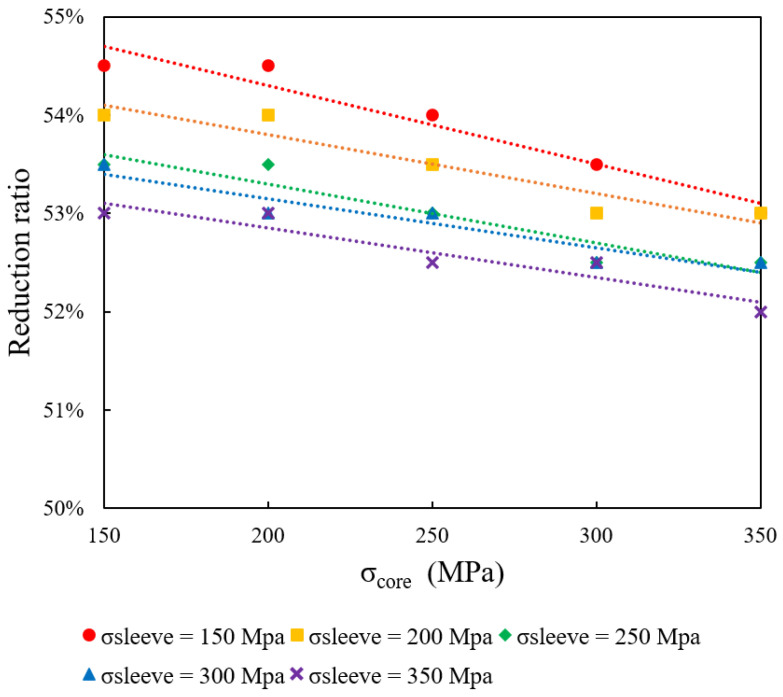
Effect of core and sleeve yield stress on maximum reduction ratio.

**Figure 3:**
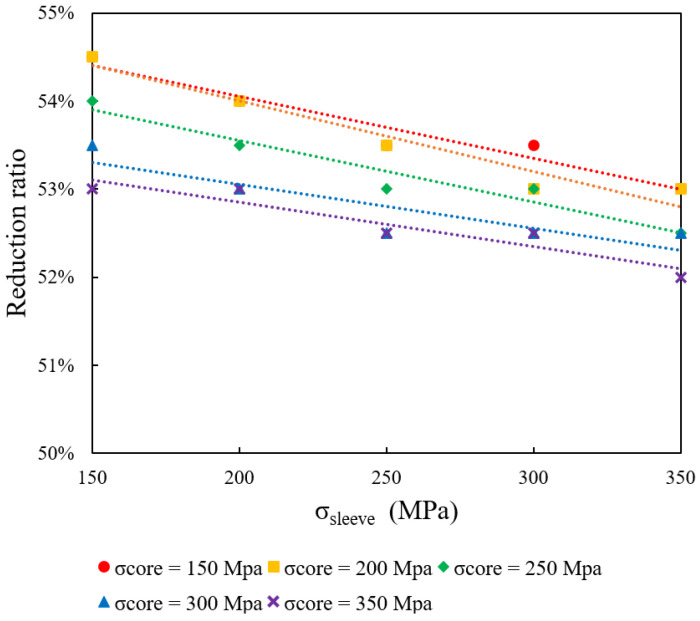
Effect of core and sleeve yield stress on maximum reduction ratio.

Table 3, Table 4, Table 5, Table 6 and Table 7 summarize the simulation outcomes for different σ_core–_σ_sleeve_ combinations, where “S” indicates successful forming and “F” denotes failure. Figure 2 and Figure 3 illustrate these results: each data point represents the maximum reduction ratio achieved under a specific set of yield stress conditions. The region below the plotted curves defines the “safe zone” (successful forming), whereas the area above corresponds to the “failure zone”. The results reveal that increasing σ_core_ reduces the maximum achievable reduction ratio. Likewise, a higher σ_sleeve_ decreases the drawing limit.

### 4.2. Impact of Initial Core Ratio on Drawing Limit at Constant Sleeve Yield Stress (250 MPa)

**Figure 4:**
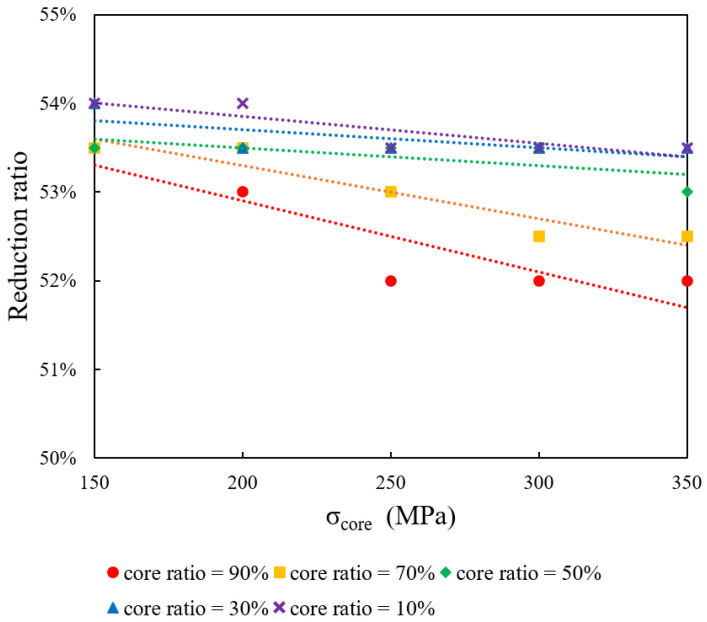
Impact of initial core ratio on formability at constant sleeve yield stress (250 MPa).

In this section, the impact of varying the initial core ratio on formability is investigated while maintaining a constant sleeve yield stress (σ_sleeve_) of 250 MPa. The core yield stress (σ_core_) is varied among 150, 200, 250, 300, and 350 MPa, and simulations are conducted for initial core ratios of 10%, 30%, 50%, 70%, and 90%. Figure 4 plots the maximum reduction ratio against σ_core_ for the different core ratios. The results consistently show that higher σ_core_ values reduce the maximum reduction ratio.

### 4.3. Impact of Initial Core Ratio on Drawing Limit at Constant Core Yield Stress (250 MPa)

**Figure 5:**
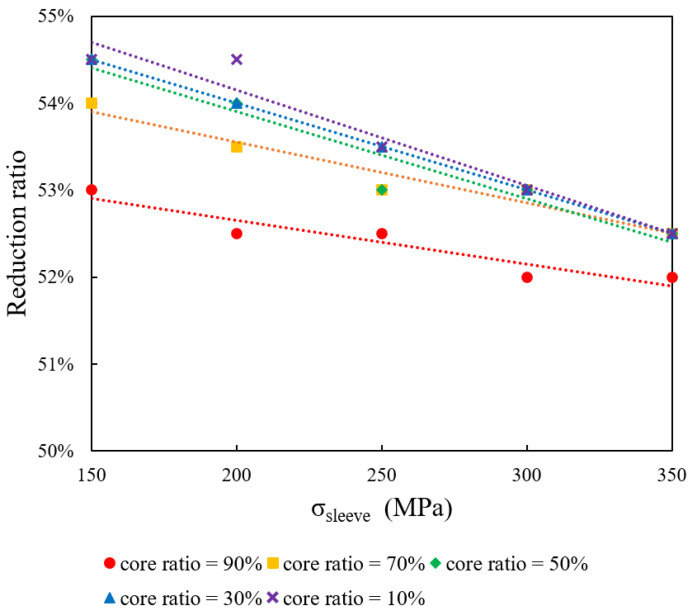
Impact of initial core ratio on formability at constant core yield stress (250 MPa).

This section focuses on the influence of the initial core ratio on the formability of bimetallic rods when the core yield stress (σ_core_) is held constant at 250 MPa. The sleeve yield stress (σ_sleeve_) is varied at 150, 200, 250, 300, and 350 MPa, while initial core ratios of 10%, 30%, 50%, 70%, and 90% are examined. Figure 5 displays the maximum reduction ratio as a function of σ_sleeve_ for the different core ratios. The data indicate that increasing σ_sleeve_ results in a lower drawing limit. 

## 5. Discussion

### 5.1. Influence of Core and Sleeve Yield Stress on Drawing Limit at a Fixed 70% Core Ratio

The analysis confirms that increasing the σ_core_ reduces the maximum achievable reduction ratio, aligning with previous findings on strain localization and premature failure in bimetallic rods. Recent studies have further explored this phenomenon, revealing additional details on interfacial stress accumulation and failure initiation mechanisms. Raghebi et al. [15] found that when the core yield stress is significantly higher than that of the sleeve, excessive stress accumulates at the core-sleeve interface, leading to early delamination. This aligns with our observation that a stiffer core limits the overall strain accommodation capacity of the structure, increasing susceptibility to interfacial failure. Their work also highlights that a high-strength core constrains deformation, concentrating strain within the sleeve, which can lead to localized necking and eventual sleeve rupture. Keller et al. [19] provided further insights by examining the microstructural evolution of bimetallic rods under different yield stress conditions. Their findings indicate that excessive yield stress contrast between the core and sleeve disrupts strain compatibility, causing microvoid nucleation at the interface. This microstructural incompatibility, in turn, accelerates fracture propagation. Our results support this conclusion, as simulations reveal that higher core yield stress increases the likelihood of brittle failure at the interface. Song et al. [20] studied Cu-Al bimetallic wires and found that the yield stress of the sleeve also plays a crucial role in determining the formability limit. Their findings indicate that a higher σ_sleeve_ increases overall resistance to plastic deformation, thereby intensifying stress concentrations at the interface. This aligns with our findings that increasing sleeve yield stress reduces formability, as the sleeve material is unable to accommodate deformation, leading to early structural failure. When both σ_core_ and σ_sleeve_ increase simultaneously, Alberti et al. [21] demonstrated that this mechanical mismatch exacerbates interfacial stress and reduces the drawing limit even further. Their study on bimetallic rods highlights the importance of achieving a balanced core-to-sleeve strength ratio, a finding that strongly correlates with our observations. Optimizing material strength ratios, combined with proper lubrication strategies, could mitigate these failure mechanisms.

### 5.2. Impact of Initial Core Ratio on Formability and Drawing Limit

The influence of the initial core ratio on drawing performance remains a critical factor in bimetallic rod processing. New studies reinforce the impact of core-to-sleeve ratios on strain distribution, fracture behavior, and interfacial integrity. Haddi et al. [23] provided numerical simulations demonstrating that excessively high core ratios (≥70%) severely restrict strain distribution. Their study observed that a high core ratio leads to a reduced sleeve thickness, which significantly weakens the outer layer’s ability to accommodate deformation. Our findings are in agreement, as simulations indicate that increasing the core ratio beyond 70% results in premature sleeve rupture due to excessive strain localization. Conversely, Roh et al. [24] explored the effects of low core ratios (≤30%) and found that such configurations tend to increase sleeve stability but at the cost of a mechanically vulnerable core. Their results indicate that when the core is too thin, strain accumulates disproportionately, increasing the likelihood of failure within the core itself. Our simulations also highlight this behavior, showing that an underdeveloped core struggles to support structural integrity under high deformation loads. Dai et al. [16] suggested that atomic diffusion at the core–sleeve interface plays a significant role in bond strength, particularly in cases where core ratios vary significantly. Their work emphasizes the necessity of optimizing annealing and surface treatment processes to improve adhesion at the interface. These findings indicate that manufacturing methods that enhance interfacial bonding could counteract some of the negative effects observed in extreme core-to-sleeve ratio configurations.

The failure mechanisms in bimetallic rods with varying core ratios can be categorized into three distinct modes. At low core ratios (≤30%), the sleeve bears most of the deformation load, leading to a more uniform strain distribution across the cross-section. However, the thin core becomes structurally weak and may experience premature failure due to excessive thinning, particularly at high reduction ratios. In contrast, when the core ratio is balanced (~50%), strain is more evenly distributed between the core and sleeve, allowing a higher reduction ratio before failure occurs. This configuration minimizes strain incompatibility and reduces the likelihood of interfacial delamination, though it requires a strong interfacial bond to prevent failure under high deformation levels. At high core ratios (≥70%), the core material dominates the structure, severely restricting the ability of the sleeve to absorb strain. In this case, high interfacial stress accumulates, leading to delamination or premature fracture at the interface. The sleeve often experiences localized necking, while the core remains largely undeformed, which ultimately results in early failure. These findings are in agreement with Szulc et al. [8], who found that composite wire drawing benefits from an optimized core-to-sleeve ratio that balances mechanical strength and formability.

The observed trends emphasize the importance of carefully optimizing the core-to-sleeve ratio and material properties to achieve both structural integrity and high formability. While a higher core ratio may enhance mechanical performance, it significantly limits deformation capacity, making the rod more prone to fracture. Similarly, increasing sleeve strength enhances durability but simultaneously reduces the ability of the sleeve to deform, increasing interfacial stress and failure risks. A moderate core ratio (~50%) appears to provide the best balance, allowing higher reduction ratios while minimizing strain localization and interfacial failure risks.

These results emphasize that yield stress variations and core-to-sleeve ratios must be carefully balanced to maximize formability while ensuring mechanical integrity. A moderate core ratio combined with optimized material properties offers the best compromise, enabling higher reduction limits while minimizing the risk of early failure.

### 5.3. Influence of Initial Core Ratio on Strain Distribution and Fracture Mechanisms

To further investigate the influence of the core ratio on the fracture behavior of bimetallic rods, simulations conducted with both σ_core_ and σ_sleeve_ equal to 250 MPa at a reduction of 54% with three different initial core ratios (90%, 50%, and 10%) are studied and coppered. 

**Figure 6:**
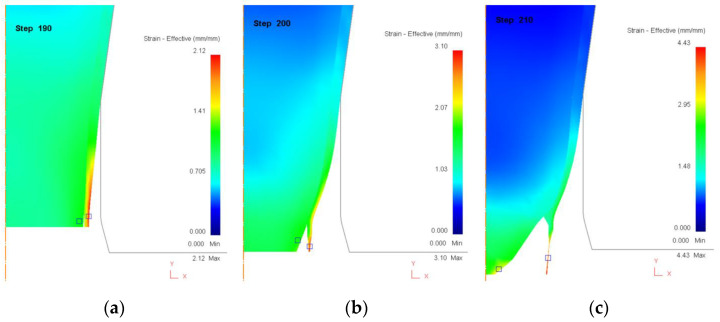
Distribution of effective strain (mm/mm) during the drawing process when core ratio is 90%. (**a**) Step 190. (**b**) Step 200. (**c**) Step 210.

Figure 6 shows the effective strain distribution during the drawing process when the core ratio is 90%. In Figure 6a (Step 190), the drawing process has progressed to a stage where initial deformation occurs primarily within the sleeve, as indicated by the localized strain concentration near the interface. The core remains largely intact, while the sleeve starts experiencing gradual plastic deformation, particularly along its outer edges. At this stage, the interfacial stress is increasing, but no visible separation between the core and sleeve has occurred yet. In Figure 6b (Step 200), the deformation intensifies, and the strain concentration within the thin sleeve layer reaches a critical level. As the sleeve material stretches beyond its plastic limit, it begins to separate from the core, indicating the onset of delamination and interfacial failure. The strain gradient is more pronounced, with high-strain zones (yellow and red regions) forming along the sleeve, signaling structural weakening and impending rupture. In Figure 6c (Step 210), the failure mechanism reaches its final stage, where complete rupture of the sleeve occurs due to excessive localized strain and necking. The core, which remains relatively undeformed, is now completely exposed as the sleeve fractures and separates. The strain distribution reveals that the sleeve cannot sustain further deformation, confirming that a 90% core ratio leads to early sleeve failure due to insufficient material thickness to support high tensile loads. This highlights the critical role of core-to-sleeve ratio in determining the fracture behavior in bimetallic rod drawing.

**Figure 7:**
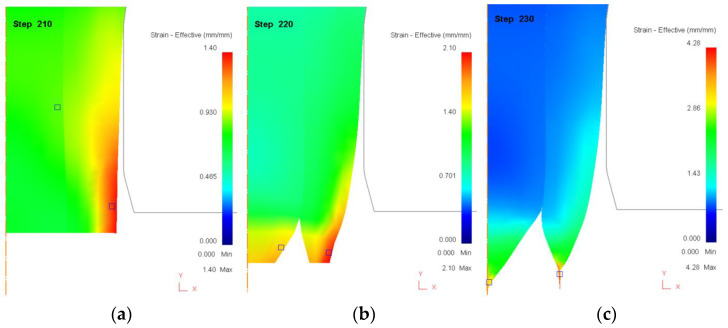
Distribution of effective strain (mm/mm) during the drawing process when core ratio is 50%. (**a**) Step 210. (**b**) Step 220. (**c**) Step 230.

Figure 7 presents the effective strain distribution for a core ratio of 50%, where the core and sleeve contribute equally to the total thickness. In Figure 7a (Step 210), the deformation process has progressed steadily, and strain distribution appears relatively uniform across both the core and sleeve. Unlike the high-core-ratio case, where the sleeve experiences excessive strain concentration, here the core and sleeve share the deformation load more evenly. The strain is primarily concentrated near the outer surface of the sleeve and at the core–sleeve interface, but no visible signs of failure are yet present. This balanced stress distribution allows the material to sustain higher reduction ratios before failure initiation. In Figure 7b (Step 220), localized strain concentration begins to emerge in both the core and sleeve, particularly near the lower central region of the rod. The presence of high-strain zones (yellow and red regions) indicates that both layers are experiencing synchronized deformation, rather than a single layer bearing the majority of the load. This marks a key difference from the 90% core ratio case, where failure was dominated by sleeve rupture. As the drawing process advances, the strain compatibility between the core and sleeve becomes increasingly critical, with the potential for interfacial shear stress to build up, leading to the early stages of delamination or necking. In Figure 7c (Step 230), the material reaches its final stage before failure, with pronounced necking visible in both the core and sleeve. Unlike the 90% core ratio case, where failure was concentrated entirely in the sleeve, here, both layers exhibit synchronized strain localization, leading to a dual-necking failure mode. This suggests that the 50% core ratio configuration supports a higher reduction limit before failure, as the load is more evenly distributed across both layers. However, if interfacial bonding strength is insufficient, delamination could still occur at this stage, compromising the structural integrity of the rod.

These observations confirm that a balanced core ratio (50%) provides improved formability compared to extreme core ratios, delaying failure and allowing for higher deformation limits. However, this configuration also demands strong interfacial bonding, as both layers experience simultaneous strain concentration. If interfacial adhesion is weak, delamination can occur even before necking leads to complete material failure, emphasizing the importance of material compatibility and bonding techniques in bimetallic rod drawing.

**Figure 8:**
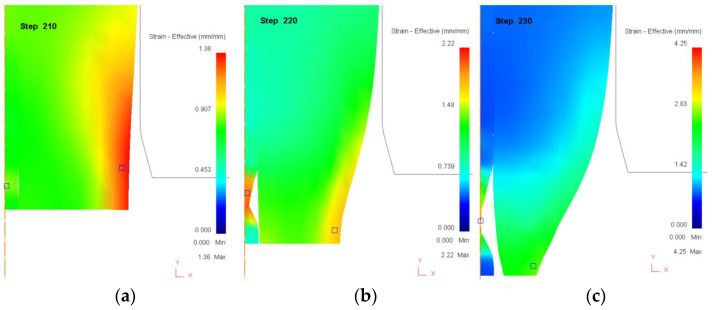
Distribution of effective strain (mm/mm) during the drawing process when core ratio is 10%. (**a**) Step 210. (**b**) Step 220. (**c**) Step 230.

Figure 8 depicts the effective strain distribution for a core ratio of 10%. In this scenario, the thicker sleeve provides enhanced structural support and distributes the applied stress more evenly across the interface. In Figure 8a (Step 210), the drawing process progresses with initial strain accumulation concentrated within the core. The thicker sleeve provides substantial structural support, allowing for a relatively uniform strain distribution. The core, being significantly thinner than the sleeve, experiences higher stress per unit area, and deformation begins earlier in the core than in the sleeve. At this stage, strain levels remain moderate, as indicated by the green-yellow coloration, and no significant necking or fracture initiation is observed. In Figure 8b (Step 220), strain concentration within the core increases significantly, with localized high-strain regions (yellow-red zones) developing along its length. The sleeve, while still providing support, begins to experience minor deformation along the interface, though the strain gradient remains more pronounced in the core. As the deformation progresses, the core reaches its plastic limit, triggering localized thinning. This marks the onset of necking, a precursor to structural failure. Unlike in the 90% core ratio case (Figure 6), where sleeve failure dominated, here the core is the critical failure site due to its reduced cross-sectional thickness and inability to sustain further plastic deformation. In Figure 8c (Step 230), the final stage of failure occurs, where the core undergoes complete localized necking and eventual rupture. The strain levels exceed critical values, reaching extreme deformation near the failure zone. The sleeve, while remaining largely intact, experiences increasing interfacial stress as the core fails, potentially leading to delamination at the core–sleeve boundary. Since the sleeve is significantly thicker than the core, it remains structurally stable, but the premature failure of the core compromises the overall integrity of the bimetallic rod. This confirms that a low core ratio (10%) results in early failure, as the thin core cannot sustain the deformation load, even if the sleeve remains structurally sound.

These findings illustrate the importance of optimizing the core-to-sleeve ratio to balance load distribution and structural stability. While a thicker sleeve provides external support, it cannot compensate for a core that is too thin to withstand drawing stresses. This failure mode differs from high-core-ratio cases, where the sleeve is the limiting factor. In contrast, a low core ratio leads to core-driven failure, restricting the maximum achievable reduction ratio.

**Figure 9:**
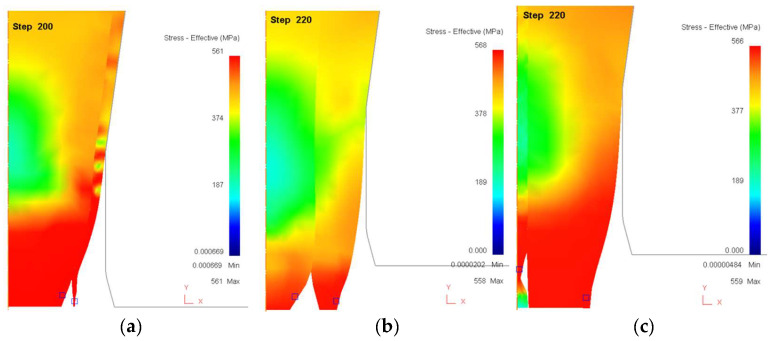
Distribution of effective stress (MPa) during the drawing process when the rods are breaking. (**a**) Core ratio = 90%. (**b**) Core ratio = 50%. (**c**) Core ratio = 10%.

Figure 9 shows the distribution of effective stress during the final stages of the drawing process, when the rods experience failure. The results highlight key differences in how stress is concentrated under varying core ratios. For Figure 9a (Core Ratio = 90%), the stress concentration is highly localized at the core–sleeve interface, particularly near the lower portion of the rod. The high stiffness of the core severely restricts the sleeve’s ability to deform, leading to excessive stress accumulation at the interface. This results in early delamination, as the sleeve cannot accommodate the required strain. Additionally, the lack of sufficient sleeve deformation makes the failure mode brittle in nature, where cracks propagate rapidly through the sleeve. In Figure 9b (Core Ratio = 50%), a more balanced distribution of stress is observed. Both the core and sleeve contribute to strain absorption, which delays the onset of failure. Although localized stress regions are still present, they are more uniformly distributed along the structure, reducing the risk of immediate interfacial delamination. This configuration allows for improved formability compared to high-core-ratio cases, confirming the advantages of an optimized core-to-sleeve ratio. For Figure 9c (Core Ratio = 10%), the core is significantly thinner, which shifts the majority of the stress concentration toward the core itself. The sleeve remains largely intact, but the core experiences extreme tensile stress, leading to failure within its structure. This highlights that while a thin core allows greater sleeve deformation, it becomes the primary failure point when subjected to high loads. The comparison of Figure 9a–c underscores the importance of achieving an optimal balance between core and sleeve properties. Extreme core ratios lead to localized stress accumulation and early failure, whereas a balanced configuration provides better stress distribution and extended drawability limits.

## 6. Conclusions

This study utilized finite element simulations to examine how variations in core and sleeve yield stress, along with changes in the initial core ratio, influence the formability, bondability, and fracture behavior of bimetallic rods during the drawing process. The findings demonstrate that higher yield stress values in either the core or sleeve significantly reduce the drawing limit, increasing interfacial stress concentrations and weakening bondability. Additionally, the initial core ratio strongly dictates deformation mechanisms, where extreme ratios (either too high or too low) lead to premature failure, while a balanced configuration (~50% core ratio) promotes better strain distribution and improved structural integrity.

The results emphasize that optimizing the drawing process requires a careful balance between material properties and geometric design. By adjusting the yield stress values and selecting an appropriate core-to-sleeve ratio, manufacturers can enhance formability, bondability, and failure resistance in bimetallic rod production. These insights provide valuable guidelines for improving industrial manufacturing processes, and future studies should focus on experimental validation and the influence of additional processing factors, such as temperature and friction conditions.

## Figures and Tables

**Table 1:** Forming parameters in finite element simulations.

Forming Parameters	Value
Initial core ratio, R_ci_/R_i_ × 100, %	10, 30, 50, 70, 90
Reduction ratio, r = (R_i_^2^ − R_o_^2^)/R_i_^2^ × 100, %	50.0, 50.5, 51.0, 51.5, 52.0, 52.5, 53.0, 53.5, 54.0, 54.5, 55.0
Friction coefficient, µ	0.1
Semi-die angle, α [°]	8
Drawing speed, v [mm/s]	10

**Table 2:** Material properties in finite element simulations.

Forming Parameters	Value
Yield stress of the core, σ_core_, MPa	150, 200, 250, 300, 350
Yield stress of the sleeve, σ_sleeve_, MPa	150, 200, 250, 300, 350
Young’s modulus of the core, GPa	207
Young’s modulus of the sleeve, GPa	207

**Table 3:** Simulation results for different σ_core_ and σ_sleeve_ combinations (σ_sleeve_ fixed at 150 MPa).

σ_y_ of Sleeve (MPa)	σ_y_ of Core (MPa)	σ_y_ Ratio (σ_sleeve_:σ_core_)	50.0%	50.5%	51.0%	51.5%	52.0%	52.5%	53.0%	53.5%	53.5%	54.0%	55.0%
150	150	1.00	S	S	S	S	S	S	S	S	S	S	F
150	200	0.75	S	S	S	S	S	S	S	S	S	S	F
150	250	0.60	S	S	S	S	S	S	S	S	S	F	F
150	300	0.50	S	S	S	S	S	S	S	S	F	F	F
150	350	0.43	S	S	S	S	S	S	S	F	F	F	F

**Table 4:** Simulation results for different σ_core_ and σ_sleeve_ combinations (σ_sleeve_ fixed at 200 MPa).

σ_y_ of Sleeve (MPa)	σ_y_ of Core (MPa)	σ_y_ Ratio (σ_sleeve_:σ_core_)	50.0%	50.5%	51.0%	51.5%	52.0%	52.5%	53.0%	53.5%	53.5%	54.0%	55.0%
200	150	1.33	S	S	S	S	S	S	S	S	S	F	F
200	200	1.00	S	S	S	S	S	S	S	S	S	F	F
200	250	0.80	S	S	S	S	S	S	S	S	F	F	F
200	300	0.67	S	S	S	S	S	S	S	F	F	F	F
200	350	0.57	S	S	S	S	S	S	S	F	F	F	F

**Table 5:** Simulation results for different σ_core_ and σ_sleeve_ combinations (σ_sleeve_ fixed at 250 MPa).

σ_y_ of Sleeve (MPa)	σ_y_ of Core (MPa)	σ_y_ Ratio (σ_sleeve_:σ_core_)	50.0%	50.5%	51.0%	51.5%	52.0%	52.5%	53.0%	53.5%	53.5%	54.0%	55.0%
250	150	1.67	S	S	S	S	S	S	S	S	F	F	F
250	200	1.25	S	S	S	S	S	S	S	S	F	F	F
250	250	1.00	S	S	S	S	S	S	S	F	F	F	F
250	300	0.83	S	S	S	S	S	S	F	F	F	F	F
250	350	0.71	S	S	S	S	S	S	F	F	F	F	F

**Table 6:** Simulation results for different σ_core_ and σ_sleeve_ combinations (σ_sleeve_ fixed at 300 MPa).

σ_y_ of Sleeve (MPa)	σ_y_ of Core (MPa)	σ_y_ Ratio (σ_sleeve_:σ_core_)	50.0%	50.5%	51.0%	51.5%	52.0%	52.5%	53.0%	53.5%	53.5%	54.0%	55.0%
300	150	2.00	S	S	S	S	S	S	S	S	F	F	F
300	200	1.50	S	S	S	S	S	S	S	F	F	F	F
300	250	1.20	S	S	S	S	S	S	S	F	F	F	F
300	300	1.00	S	S	S	S	S	S	F	F	F	F	F
300	350	0.86	S	S	S	S	S	S	F	F	F	F	F

**Table 7:** Simulation results for different σ_core_ and σ_sleeve_ combinations (σ_sleeve_ fixed at 350 MPa).

σ_y_ of Sleeve (MPa)	σ_y_ of Core (MPa)	σ_y_ Ratio (σ_sleeve_:σ_core_)	50.0%	50.5%	51.0%	51.5%	52.0%	52.5%	53.0%	53.5%	53.5%	54.0%	55.0%
350	150	2.33	S	S	S	S	S	S	S	F	F	F	F
350	200	1.75	S	S	S	S	S	S	S	F	F	F	F
350	250	1.40	S	S	S	S	S	S	F	F	F	F	F
350	300	1.17	S	S	S	S	S	S	F	F	F	F	F
350	350	1.00	S	S	S	S	S	F	F	F	F	F	F

## Data Availability

The original contributions presented in this study are included in the article, further inquiries can be directed to the corresponding author.
